# Editorial: Language Development in the Digital Age

**DOI:** 10.3389/fnhum.2017.00447

**Published:** 2017-09-05

**Authors:** Mila Vulchanova, Giosuè Baggio, Angelo Cangelosi, Linda Smith

**Affiliations:** ^1^Language Acquisition and Language Processing Lab, Department of Language and Literature, Norwegian University of Science and Technology Trondheim, Norway; ^2^School of Computing and Mathematics, Plymouth University Plymouth, United Kingdom; ^3^Department of Psychological and Brain Sciences, Indiana University Bloomington Bloomington, IN, United States

**Keywords:** language development, digital technology, learning, human-robot interaction, interdisciplinary approaches

## Introduction

The digital age is changing our children's lives and childhood dramatically. New technologies transform the way people interact with each other, the way stories are shared and distributed, and the way reality is presented and perceived. Parents experience that toddlers can handle tablets and apps with a level of sophistication the children's grandparents can only envy. In Great Britain, a recent survey of preschoolers shows that a rising number of toddlers are now put to bed with a tablet instead of a bedtime story. In the USA, a telephone survey of 1,009 parents of children aged 2–24 months (Zimmerman et al., 2007a) documents that by 3 months of age, about 40% of children regularly watched television, DVDs or videos, while by 24 months the proportion rose to 90%. Moreover, with the advance and exponential use of social media, children see their parents constantly interacting with mobile devices, instead of with people around them. Still, research in the US indicates that assistive social robots seem to have a favorable effect on children's language development (Westlund et al.).

Existing theories of language acquisition emphasize the role of language input and the child's interaction with the environment as crucial to language development. From this perspective, we need to ask: What are the consequences of this new digital reality for children's acquisition of the most fundamental of all human skills: language and communication? Are new theories needed that can help us understand how children acquire language? Do the new digital environment and the new ways of interaction change the way languages are learned, or the quality of language acquisition? Is the use of new media beneficial or harmful to children's language and cognitive development? Can new technologies be tailored to support child growth and, most importantly, can they be designed to enhance language learning in vulnerable children?

These questions and issues can only be addressed by means of an interdisciplinary approach that aims at developing new methods of data collection and analysis in a longitudinal perspective. This type of research is however not yet documented.

### Past and current research

The question of how the ecology of the child affects the acquisition of competencies and skills has been approached from different perspectives in different disciplines. In linguistics, the central question addressed concerns the specific role of exposure to language. Two influential types of theory have been proposed. One view is that the capacity to learn language is hard-wired in the human brain (Chomsky, 1965; Pinker, 1994); linguistic input is merely a trigger for language to develop. From an alternative view, language acquisition depends on the linguistic environment of the child, and specifically on language input provided through child-adult communication and interaction (Tomasello, 2003). The latter view further specifies that factors in interaction are crucial for language learning to take place. Such views are aligned with overarching theories of human development in cognitive science and psychology. These theories (known as embodied and situated cognition theories) hold that knowledge is acquired by humans through rich physical and social interaction with their environment (Barsalou, 2008). This interaction leaves multiple traces provided by a number of modalities (auditory, visual, haptic etc.) and helps consolidate knowledge in the brain by strengthening the neural networks that support learning and the use of knowledge. Exactly how input received from multiple, and multi-sensory in nature sources, interacts in both knowledge acquisition and use is, however, still poorly understood.

A current theme in the fields of information technology, artificial intelligence and robotics is to create robots that develop, as children do, and to establish how embodiment and interaction support language learning in these machines. These artificial models will eventually inform us about child development and vice versa (Cangelosi and Schlesinger, 2015, forthcoming). In the field of human-machine interaction, research is investigating whether using a physical robot, rather than a virtual agent or a computer-based video, has a positive effect on language development. Kennedy et al. (2015), for example, investigate how toy-like robots, such as, the Aldebaran Nao, are used in the classroom instead of, or together with, digital tools such as tablets, to show how a richer embodied technology method further improves language learning. Vogt envisage that, in the digital age, social robots will increasingly be used for educational purposes, such as, second language tutoring. They propose a number of design features to develop a child-friendly social robot that can effectively support children in second language learning, and discuss the technical challenges for developing such tutors.

In education research, the main question is the extent to which the use of tablets can facilitate learning to read and write, and how this type of learning compares to traditional learning. In this context, Guerra and Mellado observe that implementing information and communication technologies for educational contexts that have robust and long-lasting effects on student learning outcomes is still a challenge. They further suggest that any such system must be theoretically motivated and designed to tackle specific cognitive skills (e.g., inference making) supporting a given cognitive task (e.g., reading comprehension), and must be able to identify and adapt to the user's profile. Furthermore, a field that combines the concerns of education and digital technology is newly emerging, where one of the questions is how games should be designed to facilitate learning. Zhang et al. provide a review of the educational application of Massive Multiple Online Role-Playing Games (MMORPGs) based on relevant macroscopic and microscopic studies, showing that gamers' overall language proficiency or some specific language skills can be enhanced by real-time online interaction with peers and game narratives or instructions embedded in the MMORPGs. Mechanisms underlying the educational assistant role of MMORPGs in second language learning are discussed from both behavioral and neural perspectives, highlighting the role of attentional bias. Child-media interaction has also been approached in psychology, raising the issue of how new technologies change behavior and interaction, including values and communication patterns.

A recurrent problem in most recent research, however, is that the topic has been approached from a single disciplinary perspective, and often with a single theory in mind. Accounts are piecemeal and explain only one phenomenon at a time. Despite considerable advances in the past 20 years, we miss a holistic model of language development that also integrates the impact of digital technology on its outcomes. Such a model must take into account the weighting of all factors involved. One major challenge is the nature and amount of data that need to be collected and analyzed to build such a model. These data are, in their nature, multi-modal, complex, and dense. It then becomes mandatory to develop new analytic methods and to integrate the complex data needed in order to answer the following three fundamental questions:

- How should traditional theories and models of language acquisition be revised to account for the multimodal and multichannel nature of language learning in the digital age?
- How should existing and future technologies be developed and transformed so as to be most beneficial for child language learning and cognition?
- Can new technologies be tailored to support child growth, and most importantly, can they be designed in order to enhance specifically vulnerable children's language learning environment and opportunities?

## First language development

### Early research on the mass media and language development

Interest in the impact of the mass media on language development started as early as the late 70-ies. One of the questions that was asked was “Does the language of the mass media contribute a “new” language compared to traditional forms of communication (e.g., books or oral language)?” It was suggested that the new mass media (film, radio, TV) offer “new” languages whose grammar was yet unknown (McLuhan, 1964; Willie, 1979), and, as such, were potentially qualitatively different form oral human-to-human communication. One specific aspect where this difference was particularly salient is the multimodal nature of media, such as television and film. It has been observed that the vehicles of messages in these media involve the marriage of two languages with completely different characteristics (auditory/oral & visual/pictures) (Willie, 1979).

Some results from this early research indicate that there are certain behavioral consequences. For instance, TV-viewing appears to lead to less reading, yet subject to individual variation (Himmelweit et al., 1960). Furthermore, TV-viewing leads to less listening to the radio, and, in particular, with more adverse effect for “brighter” children (greater loss). In contrast, a study on the popular children's programme Sesame Street found a positive effect of TV viewing on language development, however, only in combination with adult intervention (Winn, 1977). Other research suggests that TV viewing overall has a negative effect on the development of children's attention and cognition and the American Academy of Pediatrics has recommended that children below 2 years of age not watch any television (Anderson and Pempek, 2005).

A valid question if we should expect any impact of mass media on language development is the extent to which the content provided through the media is comprehensible. How much of what children view on TV do they understand? Studies have shown that comprehension tends to increase with age with only 20% understanding among 4-year olds. Also, since this kind of input is mediated through both modalities, the visual and the auditory, advance in language development ought to depend both on the child's non-verbal (visual cognition) and verbal cognitive status at point of exposure. As evidenced by the papers in the current volume, tailoring the features of the technology used to the individual level of cognitive and language skills of the learner is a major prerequisite for successful outcomes. Moreover, as argued by Acerbi, one needs to understand how cultural transmission processes (e.g., transmission biases), of which language learning is arguably one instance, function in the new context of digital media.

When comparing the effects of TV and radio exposure, there is a crucial difference between language experience that requires no reciprocal participation (radio, TV) in contrast to active exchange with another person. Furthermore, TV-images do not go through a complex symbolic transformation; the mind does not decode or manipulate information, as with other types of oral or written language input.

Later research has focused on the extent to which first language acquisition from exposure exclusively to the mass media (radio and TV) deviates from typical language acquisition through interaction with care-givers and peers. Several findings suggest that overwhelming exposure to the kind of input from the radio or TV can have adverse effects, especially for very young children (toddlers). Thus, in a longitudinal study, Zimmerman and Christakis (2005) document that early TV exposure in children younger than 3 years of age was associated with deletirious effects on cognitive development, such as reading at age 7, while infant exposure (between 8 and 16 months) to videos/DVDs was associated with a 16.99-point decrement in CDI score (Zimmerman et al., 2007b). Tanimura et al. (2007) studied 18-month old infants (*n* = 1,900) and found that those who engaged in frequent TV-viewing (>4 h per day), even when accompanied by parental talking, had delayed language development/speech production (in terms of meaningful words). An observational study of 14 pairs of children (age range 7–24 months) and parents videotaped while watching television together shows that both the quality and quantity of parental utterances (Child-directed Speech) significantly declined while the TV was on, and especially when the infants were watching. This also led to an increase of frequency of 1-word sentences, quite often only short phrases, such as nouns (names). From a broader perspective, there is evidence that educational programmes targeting infants and toddlers have not achieved their purported learning goals (cf. Hirsh-Pasek et al., 2015 for a review).

Given that what children watch is important for subsequent vocabulary development (Anderson, 1998; Linebarger and Walker, 2005), and how children watch (with parent or not) is also relevant (Jordan, 2004; Anderson and Pempek, 2005), such findings are extremely pertinent for current research to follow up on. Moreover, the results from the study by Zimmerman et al. (2007b) reporting a negative correlation between DVD viewing and vocabulary development have been challenged by a recent re-analyses of the data set from that study (Ferguson and Donnellan, 2014). This replication found that effect sizes were negligible between analyses for positive, neutral, and negative effects. Interestingly, infants exposed to no media had lower levels of language development compared to infants with some exposure. Thus, it seems that more variables are necessary to take into account in the equation.

### From TV and radio to tablets and robots

Modern digital technology has attracted the attention of scholars due to its favorable affordances. It allows for multi-sensory interaction and provides rich input in the form of visual, auditory, and haptic stimuli (Belpaeme et al., 2012). A recent study by Allen et al. (2015) exploits the multi-modal nature of the input provided by iPads. The main question addressed in that study is whether iPads might promote symbolic understanding and word learning in children with autism in comparison with age-matched typically developing controls. The hypothesis was that multiple, differently colored exemplars of target referents, as afforded by the iPad technology, might promote phonological pattern-meaning/referent associations, e.g., compared to single exemplars. The study included four conditions, contrasting the use of an iPad vs. a Book, and exposing the children to single vs. multiple exemplars of the target items. Participating children were tested on whether they would associate the word to a 3-D referent (real life object) and whether they would generalize it to another member of the same category, but shown in a different color. The results indicated no differences between the two types of media (iPad or book) in symbolic understanding and level of generalization. They further demonstrate that exposure to multiple exemplars increases the rate of extension from picture to 3-D object.

Other studies have focused on how technology can assist exposure to language through reading. Chang and Breazeal (2011) propose to combine a basic primer book with interactivity in order to support parent-child reading interactions during shared book-reading. The design targets very young children (2–5 years) and offers a variety of features: it enables physical proximity, is visually accessible, responds to touch, is navigable to both child and parent, and encourages vocal expression. One specific aspect deserves mention, the Multisensory Contextual Selections. Thus, speech and touch combine to alter the content, and the reader can change story elements using a combination of touch and speech, encouraging creativity and variation. This design is based on interviews and suggestions thereof with educational experts, designers and researchers and exploits the interactive affordances of digital technology. From the point of view of child-parent interactions, Kucirkova et al. (2014) suggest that multimedia story sharing resembles interactions similar to those when experiencing a piece of art in terms of its holistic nature. Furthermore, there is some evidence that personalization of digital multimedia formats leads to more spontaneous speech production in children (Kucirkova et al., 2014).

### Second language learning

Westlund et al. (2016) investigated the role of social robots in second language learning. The study had two main goals. The first one was to test whether a socially assistive robot could help children learn new words in a foreign language more effectively by personalizing its affective feedback. The second aim was to demonstrate the feasibility of creating and deploying a fully autonomous robotic system at a school for several months. The design included a socially assistive robotic learning companion to support English-speaking children's acquisition of a new language (Spanish). In a two-month microgenetic study, 34 preschool children played an interactive game with a fully autonomous robot and the robot's virtual sidekick, a Toucan shown on a tablet screen. Two aspects of the interaction were personalized to each child: (1) the content of the game (i.e., which words were presented), and (2) the robot's affective responses to the child's emotional state and performance. The results from the study indicate that the children learned new words and affective personalization led to greater positive responses from the children.

Vogt et al. propose a number of features for an L2 robot tutor including ways to develop the robot such that it can act as a peer to motivate the child during second language learning and build trust at the same time, while still being more knowledgeable than the child and scaffolding that knowledge in adult-like manner. The authors suggest that the first impression of the child are crucial for building trust and common ground, thus supporting child-robot interactions in the long term. Other important features relate to the ability to adapt to the language proficiency level of the individual child, respond contingently, both temporally and semantically, provide effective feedback and monitor children's learning progress, as well as establish joint attention, and use meaningful gestures. There are a number of technical challenges associated with such an optimal design, such as, automatic speech recognition (ASR) for children, reliable object recognition to facilitate semantic contingency and establishing joint attention, and developing human-like gestures with a robot that does not have the same morphology as humans. The paper presents an experiment which investigates how children respond to different forms of feedback from such a robot.

## Child-robot interaction

While we still lack in-depth longitudinal studies of the effects of current digital technologies on language learning, child-robot interaction has been studied recently. Breazeal et al. (2016) looked at children ranging from 3 to 5 years who were introduced to two anthropomorphic robots that provided them with information about unfamiliar animals. This study found that the children treated the robots as interlocutors: they supplied information to the robots and retained what the robots told them. Children also treated the robots as informants from whom they could seek information. Consistent with children's early sensitivity to an interlocutor's non-verbal signals, children were especially attentive and receptive to whichever robot displayed the greater non-verbal contingency. Selective information seeking is consistent with recent findings showing that although young children learn from others, they are selective with respect to the informants that they question or endorse.

Other research in this domain indicates that children readily treat anthropomorphic robots as social companions (Shiomi et al., 2006). Kahn et al. (2013) document that children often respond verbally to robots (beyond what one might give to an automated system). This research also shows that robots are often attributed mental attributes (emotions etc.), and further that young participants readily engage in verbal exchange with (e.g., speak to) robots.

Movellan et al. (2009) assessed learning from a robot. In that study toddlers (18–24 months) interacted with a sociable robot which displayed images of 4 objects. At pre-test the toddlers' choices were a little better than chance. Over a 2-week period a modest learning outcome was observed, in that there was a significant improvement on taught words, but no improvement on control words. Tanaka and Matsuzoe (2012) studied word learning in the context of a social robot in the age range between 3 and 6 years. The robot responded either correctly of incorrectly to test questions about the novel words. Children reacted and spoke to the robot, and tried to teach the novel words to the robot. Furthermore, they learned the meaning of some novel action words in the company of the robot. However, the results of this study remain unclear as the children's utterances were not analyzed.

All of the studies investigating Child-Robot interaction indicate that the features of the robot are important, and that children differentiate among potential informants. Thus, accent (Kintzler et al., 2011), familiarity (Corriveau and Harris, 2009), turn-taking behavior: contingent responsiveness (Murray and Trevarthen, 1985; Nadel et al., 1999) have all been implicated as central for the interaction and learning outcomes. These findings are consistent with factors in early language development. Thus, contingent responsiveness has been shown to be essential for language learning in infancy (Kuhl, 2007), even though earlier studies have suggested that children acquire native competence regardless of whether spoken to by parents or not. Still, this topic has remained largely out of the focus of current research, and the role of child-directed speech is still to be assessed. Other factors with clear impact on language development are joint attention and accompanying gestures (Tomasello, 2006; Esteve-Gibert et al., 2016). Thus, implementing those features in social robots is likely to have a positive effect on language learning as well.

## Interim summary

The current review has revealed the following findings. Children readily interact with robots. While current research has focused on child-parent interaction while engaging with tablets/iPads, as well as learning in educational contexts, little is known about interaction and language learning from digital devices when the child is the sole agent. The level and quality of interaction largely depends on robot features. As pointed out by Belpaeme et al. (2012), for robots to interact effectively with humans, they need to be capable of coordinated and timely behavior in response to social context. Moreover, they need to display adaptive behavior. Children are likely to interact and engage in verbal exchange (e.g., speak to robots), provided robots feature contingency of responses, provide effective feedback and monitor children's learning progress, as well as establish joint attention, and use meaningful gestures. Yet, very few studies document specific advances in language learning. Thus, so far we see only modest language learning and primarily restricted to vocabulary, but only in experimental settings (Westlund et al.). Nothing is known about “outside of laboratory settings.” Overall, there is almost no research on language development *per se*.

In a recent detailed review and discussion of educational apps and their affordances, Hirsh-Pasek et al. (2015) emphasize the role of experience and the environment in the process of acquiring knowledge in early development: whether involving language or not. In particular, the path from sensori-motor experience to symbolic learning, as envisaged in approaches influenced by the Piagetan tradition, appears to be of crucial importance for unpacking the impact of digital technologies on the language learning infant. Similar perspectives need to be in focus when assessing the role of tablets (iPads) in early education (Kucirkova, 2014).

## New research agenda

The study of language learning in rich environments, including digital tools, poses specific challenges to theoretical and empirical research. Traditional theories of language acquisition emphasize characteristics of the learner, such as innate structures and maturational constraints, as well as of the input (its quality, quantity, and variation), but typically they do not take into account the different *channels* through which linguistic and contextual data are provided to the learner. The standard channel is human face-to-face interaction, accompanied by books or printed or recorded material later during childhood. However, the digital age is making new channels available to children earlier on. Each such channel provides input to infants and children through multiple sensory modalities simultaneously—not just hearing, but vision, touch etc. Should empirical research show that vocabulary or grammar learning modes or outcomes vary, depending on the channels through which the linguistic input is provided to the child, theories of language acquisition would have to be expanded, so as to include explicit models of how these effects come about. In particular, *learning theories* (modeling the input and learner) should be accompanied by *transmission theories* (also modeling the input's sources and transmission channels).

Research on language development in the digital age requires us to understand better the standard modes and channels of language transmission, i.e., vertical social learning. In most modern experiments on (artificial) language learning, the learner is exposed to linguistic or related stimuli that are “produced” by machines, e.g., a computer, not by other human beings. Implicitly, much research on language learning involving exposure or training phases is already research on learning from digital tools. There is research on language learning and use in social contexts (Tomasello, 2003), however these two lines of work have not yet been integrated: what is needed are experiments in which learning from others and learning from digital tools are directly compared, i.e., where the learning channel is an explicit experimental factor. This approach may help understanding the cognitive and behavioral consequences of learning in digital ecologies, while keeping other factors under experimental control. For example, one could directly test whether digital tools are simply increasing the *amount of information* that is made available to children, or whether instead they are facilitating or impeding learning (e.g., of new vocabulary) when information quantity is held constant. The same mutatis mutandis would hold for information quality and variation. A further set of questions is whether the effects of digital tools on learning are short-lived or long-lasting, and whether they manifest themselves invariably or only early during development: would the child's brain eventually adapt to the multiplicity of channels and respective modalities through which language is experienced? Longitudinal designs are necessary to answer such questions.

The development of robot tutors to support early language development, as well as L2 language acquisition, offers innovative ways of exploiting the digital age technologies for language tutoring purposes, and in general, for child-robot interaction. Research has consistently demonstrated that the physical presence (embodiment) of a robot (e.g., Kennedy et al., 2015; Cangelosi and Schlesinger, forthcoming), as well as some of its anthropomorphic features (robot appearance with human-like shape; e.g., Walters et al., 2008) and behavior (shared gaze, gestures; e.g., Zanatto et al., 2016), improve the outcome of the tutoring and companionship objectives. Moreover, multimodal approaches to human-robot interaction, such as, those combining tablet-based interfaces with the robot's speech communication capabilities and behavioral feedback strategies, improve the acceptability and efficacy of robot companions (Belpaeme et al., 2012; Di Nuovo et al., 2016). As such, future research directions in robot tutors for language development will benefit from the investigation of hybrid robot and digital technologies, strategically exploiting the benefits from the robot's anthropomorphic features.

Robot companions also offer the opportunity to support language acquisition in children with atypical development. Pioneering studies have looked at social assistive robotics for children with autism spectrum disorder (ASD) (e.g., Dautenhahn, 1999; Scassellati et al., 2012). For example, Scassellati et al. (2012) suggest that the improvement of social skills development via robot interaction is the consequence of the fact that robots provide novel sensory stimuli to the ASD child. Robot companions have also been used for the support of children with diabetes (Belpaeme et al., 2012) and with mobility and motor disabilities (Sarabia and Demiris, 2013). Thus, future work combining robot tutors with populations with atypical cognitive and motor development will contribute to the challenges of language skills acquisition in children with disabilities.

Future research should harvest evidence of language development in interaction with digital tools (including social robots). It should compare children who are often exposed to ICT to children who are not. It should investigate how new media/digital tools impact on the development of lower level language skills (e.g., vocabulary, grammar); how new media/digital tools impact on the development of “higher” skills (e.g., discourse comprehension) and explore the development of dimensionality (Language and Reading Research Consortium, 2015), and specifically, the effect of digital technology on oral and reading comprehension, and figurative language skills. A broader and overarching issue is the effect of new digital environments on brain plasticity and learning (Bavelier et al., 2010). Future research on this topic is also in need of novel methods for data analyses.

## Author contributions

MV prepared the initial draft and worked on subsequent editing. GB and AC worked on specific sections. LS completed final editing and alignment of ideas.

### Conflict of interest statement

The authors declare that the research was conducted in the absence of any commercial or financial relationships that could be construed as a potential conflict of interest.
